# Piezo1/ITGB1 Synergizes With Ca^2+^/YAP Signaling to Propel Bladder Carcinoma Progression via a Stiffness‐Dependent Positive Feedback Loop

**DOI:** 10.1002/cam4.71059

**Published:** 2025-07-16

**Authors:** Minghai Ma, Jianpeng Li, Xing Li, Minxuan Jing, Lu Wang, Yunzhong Jiang, Zezhong Yang, Jiale He, Min Wang, Hang Liu, Yutong Chen, Kaibo Mi, Lei Wang, Jinhai Fan, Hongxia Du

**Affiliations:** ^1^ Department of Pharmacology and Toxicology School of Basic Medical Sciences, Xi'an Medical University Xi'an China; ^2^ Department of Urology The First Affiliated Hospital, Xi'an Jiaotong University Xi'an China; ^3^ Department of Thoracic Surgery Tangdu Hospital, Air Force Medical University Xi'an China

**Keywords:** calcium ion, extracellular matrix, integrin β1, Piezo1, positive feedback loop

## Abstract

**Background:**

Emerging evidence implicates mechanotransduction pathways in modulating bladder carcinoma (BLCA) pathogenesis. However, the crosstalk between Piezo1 and integrin β1 (ITGB1) in extracellular matrix (ECM) stiffness‐driven tumorigenesis remains a critical knowledge gap. This study systematically investigates the functional synergy of Piezo1/ITGB1 in orchestrating ECM biomechanical remodeling to fuel BLCA progression.

**Methods:**

Utilizing an integrative framework combining clinical histopathology, in vivo tumor models, multiomics profiling, molecular biology experiments, matrix stiffness quantification, calcium flux analysis, and YAP signaling interrogation, we dissected the mechanochemical interplay between Piezo1/ITGB1 activation and ECM dynamics.

**Results:**

Clinical and animal data revealed that Piezo1/ITGB1 coactivation was strongly correlated with ECM stiffness‐induced BLCA proliferation and poor clinical prognosis. The coordinated expression of Piezo1/ITGB1 enhanced ECM interaction, organization, adhesion, and collagen binding. Crucially, Piezo1/ITGB1 overexpression promoted cancer development by suppressing apoptosis and enhancing proliferation. Mechanistically, ECM stiffness triggered Piezo1/ITGB1‐dependent Ca^2+^ influx, which facilitated YAP nuclear translocation and subsequent upregulation of downstream targets CTGF, α‐SMA, and COL1A1, thereby reinforcing collagen deposition and matrix stiffening.

**Conclusion:**

Our work uncovers a feedforward mechano‐oncogenic axis wherein Piezo1/ITGB1 cooperativity converts ECM cues into sustained protumorigenic signaling through Ca^2+^/YAP‐mediated transcriptional reprogramming. This paradigm redefines ECM stiffness not merely as a pathological consequence but as an active driver of BLCA progression, proposing precision targeting of the Piezo1/ITGB1 triad as a mechanotherapy strategy.

## Graphical abstract

Piezo1/ITGB1 axis and Ca^2+^/YAP signaling cooperatively drive BLCA progression via ECM stiffness sensing, eventually forming a positive feedback regulation loop.

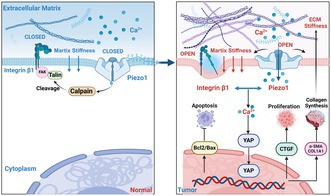

## 1 Introduction

Bladder carcinoma (BLCA) is a malignancy originating from the epithelial tissues of the bladder, causing significant patient suffering due to long‐term cystoscopy and intravesical instillation of chemical drugs [1]. The high stiffness of the bladder wall may signal advanced or muscle‐invasive BLCA, requiring total radical resection [2]. While research has indicated links between extracellular matrix (ECM) stiffness variations and BLCA development, a well‐established mechanism is lacking [3, 4, 5]. Therefore, investigating appropriate targets for estimating ECM stiffness will benefit the diagnosis and treatment in cancer management [6]. ECM is a complex network of proteins and molecules surrounding the tumor microenvironment, providing structural support and playing a crucial role in cellular processes such as tumor cell adhesion, migration, proliferation, and differentiation [7, 8]. Accumulation and stiffness of ECM can encourage cancer development, progression, metastasis, and invasion [9]. The development of solid tumors is associated with aberrant ECM deposition and remodeling of the primary microenvironment, increasing mechanical stresses sensed by tumor cells [10]. Stiffening in fibrotic tissues correlates with increased deposition of dense ECM in the lamina propria, and the increase in tissue compliance is observed before the onset and invasion of the tumor [11]. ECM may also act as a barrier to chemotherapy and immunosuppressive drugs, complicating cancer treatment. Therefore, exploring novel ways and mechanisms to target the ECM is an active area of research for potential cancer therapeutic strategies [12, 13].

Various mechanosensitive proteins, including Piezo1, the Integrin family, YAP/TAZ, and TRPV4, are primarily involved in the formation and development of ECM stiffness, working together to accelerate the synthesis and deposition of ECM components [7, 14, 15]. Piezo1, also known as FAM38A, is a mechanically activated ion channel protein implicated in the pathogenesis of various cancers [16, 17]. Piezo1 could convert mechanical stimuli into biochemical signals through mechanotransduction [18, 19]. Several studies have demonstrated that Piezo1 is upregulated in multiple types of cancer, including breast, liver, and lung cancer [20, 21]. Besides, integrins, as the primary mediators of ECM, are a family of heterodimeric transmembrane receptors composed of α and β subunits that play a pivotal role in ECM development [22]. Through their association with ECM proteins, integrins modulate cellular behavior by regulating adhesion, migration, and differentiation [23]. Integrins also play a crucial role in mechanotransduction, influencing cellular signaling pathways like focal adhesion kinase (FAK), Src, and PI3K/Akt [24, 25]. Reports have shown that integrin β1 (ITGB1) can interact with piezo1 and be feed‐forward to upregulate Piezo1 function in cardiac fibrosis. However, the relationship between Piezo1/ITGB1 and ECM stiffness in cancer is not clear [26].

Herein, we utilized bioinformatics analysis, tissue chips, molecular biology methods, matrix stiffness assays, and animal experiments to explore the function of the mechanical axis Piezo1/ITGB1/YAP in BLCA development and ECM remodeling, aiming to search for a promising way and novel tactic for BLCA management.

## 2 Materials and Methods

### 2.1 Materials and Regents

Oligonucleotides and primers were custom synthesized by Shanghai Sangon Biotech Co. Ltd., with sequences detailed in Table S1. The PrimerScript RT reagent kit and SYBR Green Master Mix were procured from TAKARA Biotechnology Co. (Dalian, China). The CCK‐8 reagent was sourced from TargetMol (USA). Agonists (Yoda1) and inhibitors (GsMTx4) for Piezo1 were acquired from MedChemExpress (China). The small interfering RNA (si‐RNA) against Piezo1 and ITGB1 was synthesized by Genepharma Technology. Overexpression plasmids for ITGB1 and Piezo1 were synthesized by Biokeeper (China). Antibodies specific for ITGB1, Piezo1, YAP1, COL1A1, CTGF, α‐Tubulin, and β‐Actin were purchased from Abclonal, while antibodies against Bcl2, Bax, and α‐SMA were obtained from Abcam. Cell culture medium was supplied by Procell Life Science & Technology Co. Ltd., and Cell Freezing Medium was from New Cell & Molecular Biotech Co. Ltd. For Ca^2+^ analysis, Fluo‐4AM was purchased from Beyotime Biotechnology (China). These chemicals used in the study were shown in Table S2.

### 2.2 Animals Experiment

This project was approved and supervised by the Experimental Animal Ethics Committee of Xi'an Jiaotong University (Approval Number: XJTUAE2023‐545). Five‐week‐old male nude mice were purchased from Beijing Huafukang Biotechnology Co. LTD. After a 1‐week quarantine, formal experiments were conducted. The mice were divided into three groups, with eight mice in each group, for the xenograft tumor experiments. T24‐Vector cells, Piezo1‐overexpressed cells, ITGB1‐overexpressed cells (6 × 10^6^/mL) were injected subcutaneously into the right dorsal region. The body weight and tumor size of each animal were measured every 3 days, and the total volume was calculated as V = (length × width^2^)/2. Tumors were obtained for H&E staining, immunohistochemical staining (IHC) and Masson staining when they reached an average volume of 100 mm^3^.

### 2.3 Tissue Chips

The study was conducted in accordance with the Declaration of Helsinki and approved by the Ethical Committee of First Affiliated Hospital of Xi'an Jiaotong University (Approval Number: LLSBPJ‐2023‐095). All the methods were carried out according to the relevant guidelines and regulations. Tissue chips derived from 181 patients with bladder cancer (BLCA) who underwent transurethral resection of bladder tumor (TURBt) in the First Affiliated Hospital of Xi'an Jiaotong University were employed to investigate the expression profile and clinical relevance of the Piezo1/ITGB1 axis.

The paraffin‐embedded tissue sections were sequentially dewaxed in xylene (three changes, 10 min each), rehydrated through a graded ethanol series (100%, 95%, 80%, and 70% ethanol, 5 min per step), and rinsed in distilled water. Antigen retrieval was performed by submerging slides in preheated citrate buffer (10 mM, pH 6.0) and incubating in a pressure cooker at 95°C for 20 min, followed by cooling to room temperature. Endogenous peroxidase activity was quenched with 3% H_2_O_2_ in methanol for 15 min at room temperature. Nonspecific binding was blocked by incubating sections with 5% BSA for 1 h at 37°C in a humidified chamber. Primary antibodies (1:200) were applied overnight at 4°C. After washing with PBS, sections were incubated with HRP‐conjugated secondary antibodies for 1 h at room temperature. Signal development was achieved using a DAB substrate kit with strict monitoring under a light microscope to optimize chromogen exposure (30–90 s), followed by immediate rinsing in distilled water to terminate the reaction. Counterstaining was performed with Mayer's hematoxylin for 1 min, differentiated in 1% acid alcohol, and blued in 0.2% ammonia water. Sections were dehydrated through an ascending ethanol series (70%, 80%, 95%, and 100%, 2 min each), cleared in xylene, and mounted with neutral balsam. Stained slides were imaged using a microscope, with consistent brightness/contrast settings across all samples. ImageJ software was used for batch analysis of staining intensity and positive cell percentage across multiple fields.

### 2.4 Database Analysis

TIMER, GEPIA, UALCAN, GeneMANIA websites were used in the study. This study employed the TIMER database (https://cistrome.shinyapps.io/timer/) to quantitatively analyze the infiltration levels of fibroblasts and immune cells in tumor tissues and explore the associations between target gene expression, immune cell abundance, and patient prognosis. Using GEPIA (http://gepia.cancer‐pku.cn/) to integrate TCGA data, we screened the differential expression of target genes between tumor and normal tissues, evaluated their clinical prognostic significance through survival analysis, and mined coexpression gene networks. UALCAN (http://ualcan.path.uab.edu/) was utilized for a stratified analysis of TCGA data to investigate correlations between gene expression and bladder tumor grade, stage, molecular subtypes, and methylation modifications. GeneMANIA (https://genemania.org/) integrated multiple data sources, including protein–protein interactions and coexpression, to construct functional association networks of target genes.

Additionally, single‐cell transcriptomic datasets of bladder cancer (GSE135337, comprising seven urothelial carcinoma samples and one adjacent normal sample) were retrieved from the GEO database (https://www.ncbi.nlm.nih.gov/geo/). The R package “Seurat” was applied for data preprocessing, encompassing quality control (filtering low‐quality cells and low‐expression genes), batch effect correction (data integration and batch effect removal via the “harmony” package), normalization, and scaling to ensure cross‐cell expression comparability. Dimensionality reduction and cell clustering visualization were performed using principal component analysis (PCA) and UMAP algorithms. To investigate the functional roles of Piezo1 and ITGB1‐related genes, Gene Ontology (GO) and Kyoto Encyclopedia of Genes and Genomes (KEGG) enrichment analyses were conducted using the “clusterProfiler” package, complemented by Gene Set Enrichment Analysis (GSEA) with “hallmark gene sets” to elucidate their involvement in biological processes.

All publicly available datasets (TCGA, GEO) used in this study were accessed in accordance with the data usage policies and ethical guidelines of their respective sources. These datasets are deidentified and comply with ethical standards for secondary analysis, as outlined by the original providers.

### 2.5 Cell Culture

The human bladder cancer cell lines J82, 5637, T24, and 253J, and the normal control cell SV‐HUC, which is the SV‐40 immortalized human uroepithelial cell line, were obtained from the American Type Culture Collection (ATCC, USA). J82 and 5637 cells were cultured in RPMI 1640 medium supplemented with 10% FBS under a humidified 5% CO_2_ and 95% air atmosphere at 37°C. T24, 253J, and SV‐HUC cells were cultured in DMEM medium supplemented with 10% FBS under a humidified 5% CO_2_ and 95% air atmosphere at 37°C.

### 2.6 Transfection and Lentiviral Infection

siRNA targeting ITGB1 and Piezo1 was procured from Genepharma Technology (China). The GP‐Transfect‐Mate reagent was utilized to facilitate the transfection of siRNA into T24 and 253J cell lines. Overexpression of ITGB1 and Piezo1 was achieved using lentiviruses, which were sourced from Biokeeper Technology (China). These lentiviruses were packaged to enhance transfection efficiency, following the provided protocol. Subsequent to a 48‐h post‐transfection period, the cells were selected using puromycin and then employed for further experimental procedures.

### 2.7 Real‐Time Quantitative Polymerase Chain Reaction (PCR)

Real‐time quantitative polymerase chain reaction (RT‐qPCR) was conducted to assess the mRNA expression levels of ITGB1 and Piezo1. The SVHUC‐1, 5637, J82, T24, and 253J cell lines were plated in six‐well plates and allowed to grow in a complete growth medium overnight. Total RNA was isolated using the Fast 200 reagent, adhering to the manufacturer's instructions. This RNA was then reverse‐transcribed into complementary DNA (cDNA) using the PrimerScript RT reagent kit from Takara Bio. The relative quantification of the target gene mRNA transcripts was achieved using the SYBR Green PCR Master Mix (Takara Bio, Dalian, China) on a C1000 Thermal Cycler (Bio‐Rad Laboratories Inc., Hercules, USA). All samples were analyzed in triplicate, and the expression levels were normalized to the housekeeping gene 18S.

### 2.8 Western Blotting Assay

Total cellular proteins were extracted using RIPA lysis buffer, supplemented with protease inhibitors, phosphatase inhibitors, and 0.1 M phenylmethylsulfonyl fluoride (PMSF), all sourced from Beyotime (China). The protein extraction and subsequent Western blotting procedures were carried out according to standard protocols. After centrifugation at 13,000 ***g*** for 15 min at 4°C, the supernatant containing the total cell proteins was collected. Protein quantification was performed using the bicinchoninic acid (BCA) assay. For electrophoresis, 20 μg of the protein sample was loaded onto a 10%–15% SDS‐polyacrylamide gel and subjected to electrophoresis. The resolved proteins were then transferred onto polyvinylidene fluoride (PVDF) membranes. To minimize nonspecific binding, the membranes were blocked with 5% nonfat milk at room temperature for 2 h. Following this, the PVDF membranes were incubated with the respective primary antibodies overnight at 4°C. After three washes with Tris‐buffered saline with Tween (TBST) to remove unbound antibodies, the membranes were incubated with a horseradish peroxidase‐conjugated secondary antibody for 1 h at room temperature. The expression of the proteins was finally detected using the Enhanced Chemiluminescence (ECL) detection system from Bio‐Rad (CA, USA).

### 2.9 Apoptosis Assay

The apoptotic ratios in T24 and 253J cells where Piezo1 and ITGB1 were knocked down were determined using flow cytometry. The cells were initially washed twice with precooled phosphate‐buffered saline (PBS) and then centrifuged at 500 ***g*** for 5 min at 4°C. Following centrifugation, the cells were resuspended in 100 μL of binding buffer. This suspension was then mixed with 4 μL of Annexin V‐fluorescein isothiocyanate (FITC) and propidium iodide (PI) and incubated for 15 min in the dark. To facilitate the flow cytometric analysis, 400 μL of binding buffer was added to dilute the sample. The resulting mixture was analyzed using a flow cytometer to measure the apoptotic ratios.

### 2.10 Proliferation Assay

Cell viability was assessed using the CCK‐8 assay. The T24 cells, as well as the T24 cells overexpressing Piezo1 (T24‐Piezo1^OE^) and Integrin β1 (T24‐ITGB1^OE^), were seeded into 96‐well plates. The cells were then cultured for various durations: 0, 24, 48, and 72 h. At each time point, CCK‐8 reagent was added to each well and allowed to incubate for 3 h to facilitate the detection of cell viability. The absorbance readings, indicative of cell viability, were measured using a microplate reader. The data obtained were analyzed using GraphPad Prism software to calculate the percentage of viable cells relative to the control at each time point.

### 2.11 Preparation of Hydrogels

In the fabrication of polyethylene glycol (PEG) hydrogels, two types of eight‐arm PEG molecules are utilized [26]: eight‐arm PEG maleimide (PEG‐MAL), serving as the backbone with a molecular weight of 10 kDa, and eight‐arm PEG thiol (PEG‐SH), acting as the crosslinker with the same molecular weight. The PEG‐MAL backbone is modified with monothiolated peptides that are then crosslinked by PEG‐SH through a Michael addition reaction. PEG hydrogels modified with 1 mM Fibronectin facilitate the establishment of integrin adhesions. PEG‐MAL is dissolved in deionized water and allowed to conjugate for 1 h at 37°C. By varying the concentration of PEG‐SH used for crosslinking the hydrogel, the elastic modulus of the PEG hydrogels can be adjusted to achieve different stiffness levels: 2, 20, and 41 kPa. The hydrogels were displayed in six‐well plates and allowed to grow in a complete growth medium for 30 min. Then T24 cells were seeded onto different hydrogels for 24 h to mimic tumor‐associated ECM conditions.

### 2.12 Immunofluorescence

T24 cells, T24‐Piezo1,^OE^ and T24‐ITGB1^OE^ cells were seeded onto glass coverslips. The cells were exposed to either Piezo1 agonists (Yoda1) or Piezo1 inhibitors (GsMTx4) for a period of 4 h to modulate Piezo1 activity. Following the treatment, primary antibodies specific for ITGB1, Piezo1, YAP, and α‐SMA were diluted at a ratio of 1:200 and applied to the cells, which were then incubated in a humidified chamber overnight at 4°C to allow for antibody binding. Subsequently, the appropriate secondary antibodies were applied to the coverslips and incubated at room temperature for 1 h to bind to the primary antibodies. To visualize the nuclei, 4,6‐diamidino‐2‐phenylindole (DAPI) was added and allowed to stain the cells at room temperature for 5 min. Finally, the coverslips were mounted onto slides using an antifluorescence quenching reagent to prevent the fading of fluorescence over time. The slides were then examined under a fluorescence microscope to visualize the fluorescence signals.

### 2.13 Ca^2+^ Analysis

T24 cells, T24‐Piezo1,^OE^ and T24‐ITGB1^OE^ cells were treated with the agonists (Yoda1) or the inhibitors (GsMTx4) of Piezo1 for 4 h and washed three times with PBS. The cells were then loaded with the Fluo‐4AM fluorescent probe, a calcium‐sensitive dye, at a concentration of 2 μM. This dye was incubated with the cells at 37°C for 30 min to allow for adequate dye uptake and intracellular esterase‐mediated de‐esterification, which is necessary for the dye to bind calcium ions. Following the incubation, the cells were washed again with PBS three times to remove extracellular Fluo‐4AM. To allow the cells to recover and stabilize, they were further incubated for 20 min postwashing. The fluorescence was then measured using both fluorescence microscopy and flow cytometry.

### 2.14 Quantification and Statistical Analysis

All experiments were performed with at least three biological replicates. Data are presented as the mean values ± standard deviation (SD). To determine the statistical significance of the observed differences, we employed Student's *t*‐test for pairwise comparisons or one‐way analysis of variance (ANOVA) for multiple group comparisons, as appropriate. A *p*‐value of < 0.05 was considered to indicate statistical significance. (*p < 0.05; **p < 0.01; ***p < 0.001). All statistical analyses were conducted using the SPSS 20.0 software.

## 3 Results

### 3.1 The Expression Profile and Clinical Significance of Piezo1/ITGB1 Axis in BLCA Tissue Chips

**FIGURE 1:**
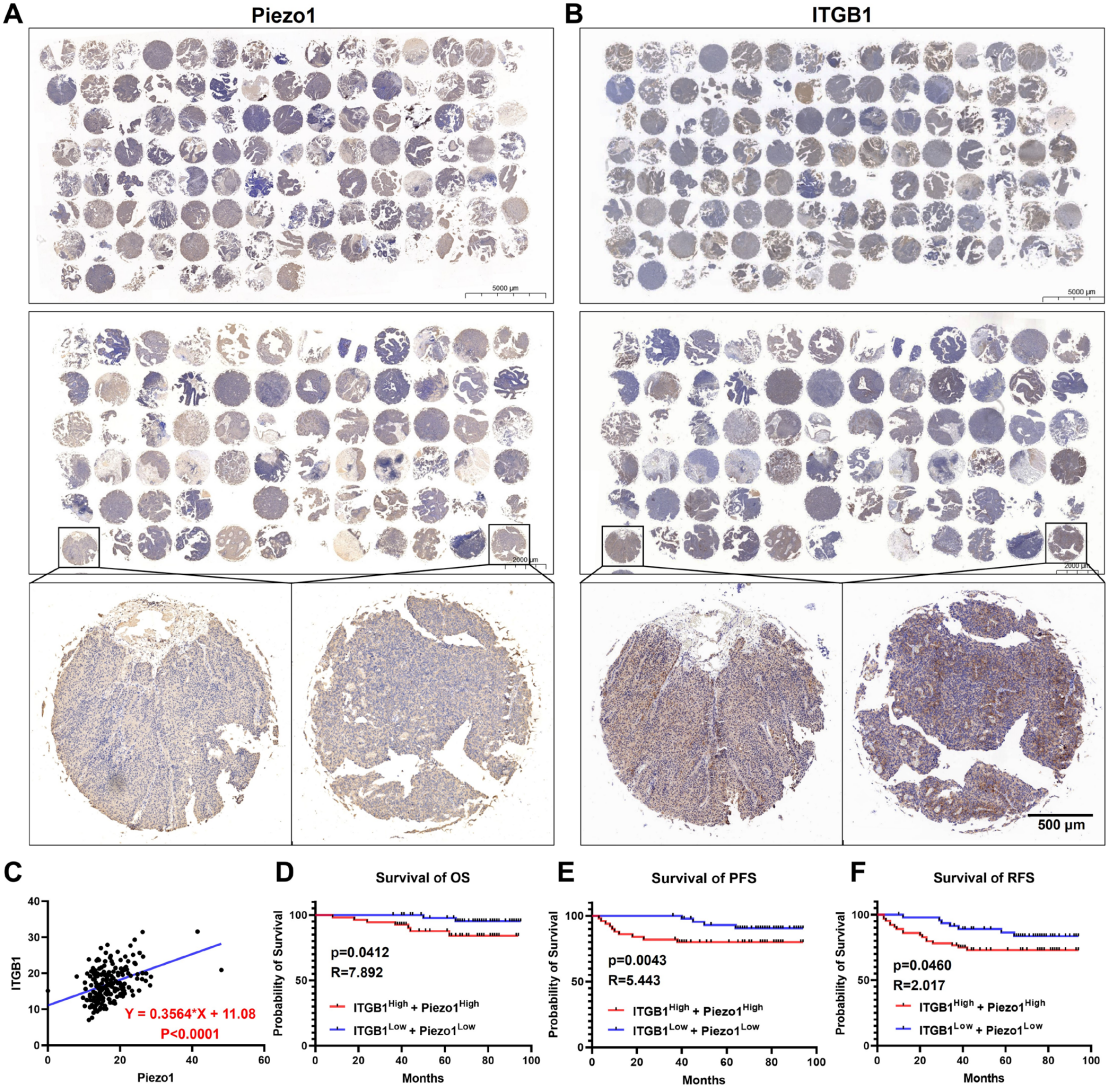
Expression profile and clinical significance of Piezo1/ITGB1 axis in BLCA tissue chips. (A) IHC expression profile of Piezo1 in BLCA chips from 181 BLCA patients who underwent transurethral resection of bladder tumor (TURBt). (B) IHC expression profile of ITGB1 in BLCA chips from 181 BLCA patients. (C) The quantitative expression level of Piezo1 and ITGB1 in BLCA tissue chips. (D) The prognosis curve for overall survival (OS) between the ITGB1^High^‐Piezo1^High^ BLCA group and the ITGB1^Low^‐Piezo1^Low^ BLCA group. (E) The prognosis curve for progression‐free survival (PFS) between the ITGB1^High^‐Piezo1^High^ BLCA group and the ITGB1^Low^‐Piezo1^Low^ BLCA group. (F) The prognosis curve for recurrence‐free survival (RFS) between the ITGB1^High^‐Piezo1^High^ BLCA group and the ITGB1^Low^‐Piezo1^Low^ BLCA group.

The tissue chips from 181 BLCA patients with T1‐stage who underwent transurethral resection of bladder tumor (TURBt) were utilized to analyze the expression profile and clinical significance of the Piezo1/ITGB1 axis. As shown in Figure 1A,B, Piezo1 and ITGB1 presented a significant upregulation in the ECM and vascular system in BLCA tissues rather than the nucleus, meaning that the Piezo1/ITGB1 axis may play a role in influencing the accumulation and deposition of ECM components. IHC results further supported a linear association between the expression levels of Piezo1 and ITGB1 (Figure 1C), with a correlation coefficient (rho) of 0.3564 (*p* < 0.0001). Additionally, patients with high expression levels of both ITGB1 and Piezo1 (ITGB1^High^‐Piezo1^High^) exhibited a poorer prognosis in terms of overall survival (OS), with a hazard ratio of 7.892 (*p* = 0.0412) (Figure 1D). Similarly, ITGB1^High^‐Piezo1^High^ BLCA patients also exhibited poorer survival than ITGB1^Low^‐Piezo1^Low^ patients for both progression‐free survival (PFS: hazard ratio = 5.443, *p* = 0.0043) and recurrence‐free survival (RFS: hazard ratio = 2.017, *p* = 0.0460), implying that the Piezo1/ITGB1 axis cooperated together in concert to accelerate the development and progression of BLCA (Figure 1E,F). The association between Piezo1 and ITGB1 expression with clinicopathologic characteristics in BLCA tissues was shown in Table S3. The expression of Piezo1 and ITGB1 was also associated with tumor size and number. In summary, the Piezo1/ITGB1 axis appeared to modulate each other's expression and potentially contribute to the accumulation of ECM, which was correlated with a poorer prognosis in BLCA patients.

### 3.2 Piezo1/ITGB1 Axis Promotes BLCA Progression and Development In Vivo

**FIGURE 2:**
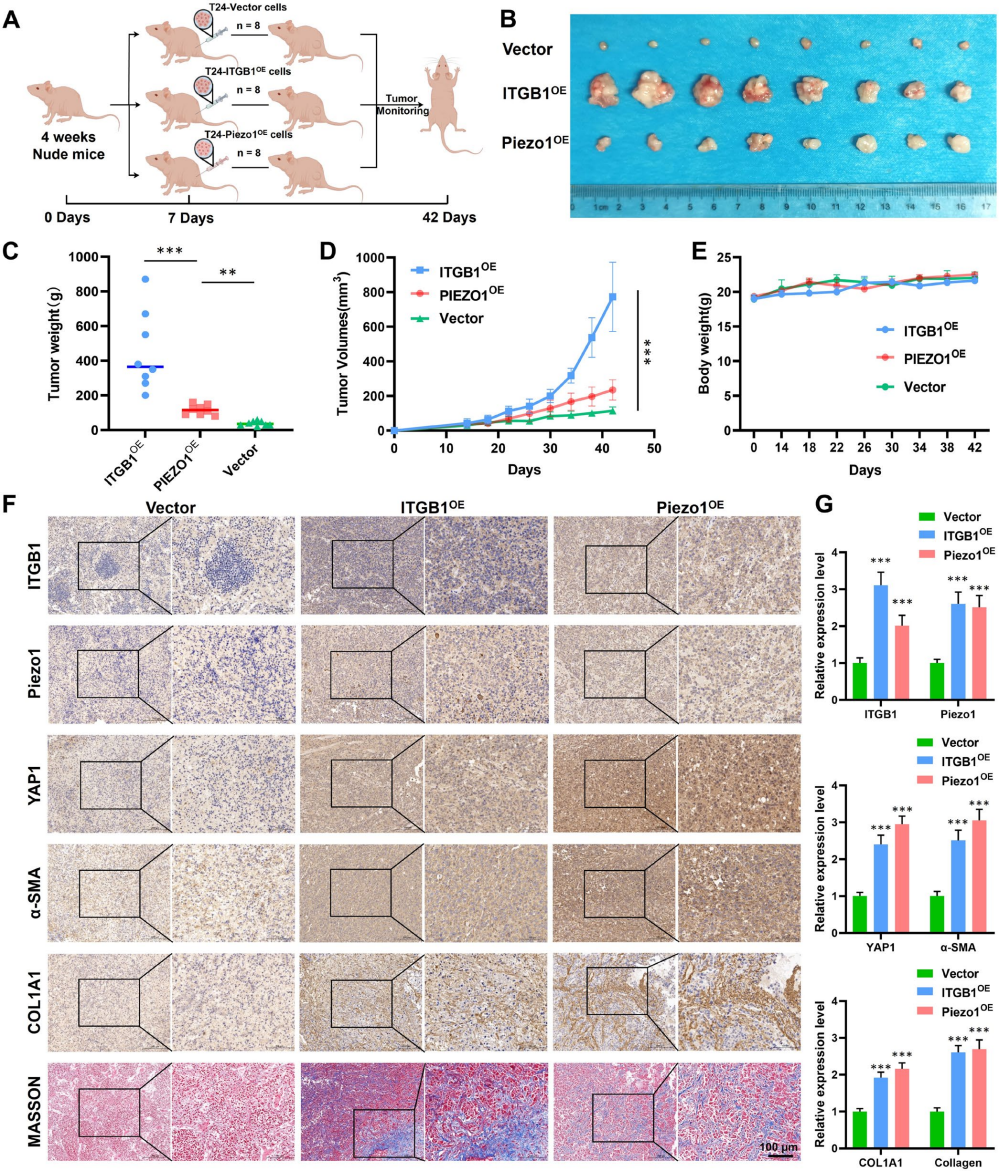
The function of Piezo1/ITGB1 axis in vivo. (A) Flow diagram of the animal experiments divided into T24‐Piezo1^OE^, T24‐ITGB1^OE^, and T24‐Vector groups. (B) Representative images of tumors harvested from the groups. (C) The tumor weights in T24‐Piezo1^OE^, T24‐ITGB1^OE^, and T24‐Vector groups. (D) The tumor volumes in T24‐Piezo1^OE^, T24‐ITGB1^OE^, and T24‐Vector groups. (E) Body weights in mice among each group. (F) Tumor tissues analyzed by H&E, IHC, and Masson staining to detect the expression of ITGB1, Piezo1, YAP1, α‐SMA, COL1A1, and collagen fibers. (G) The quantitative data of IHC and Masson staining image.

To further elucidate the role of the Piezo1/ITGB1 axis in BLCA, we conducted in vivo experiments using animal models (Figure 2A). The mice were implanted with Piezo1‐overexpressed T24 cells (T24‐Piezo1^OE^), ITGB1‐overexpressed T24 cells (T24‐ITGB1^OE^) or Vector‐transfected T24 cells (T24‐Vector). The results indicated that tumor volumes in the ITGB1^OE^ and Piezo1^OE^ groups were significantly larger than those in the Vector group (Figure 2B). Moreover, the tumor weights and volumes were significantly increased in the ITGB1^OE^ and Piezo1^OE^ groups (Figure 2C,D), while no significant changes were observed in the body weight of mice among each group (Figure 2E), further supporting the notion that the Piezo1/ITGB1 axis contributed to tumor progression. Histological analysis of the tumor tissues was performed using H&E, IHC, and Masson staining (Figure 2F). We observed significantly increased expression of ITGB1, Piezo1, YAP1, α‐SMA, and COL1A1, which contributed together to the accumulation and deposition of collagen and ECM stiffness. Simultaneously, Masson staining revealed an increase in the deposition of collagen fibers in the ITGB1^OE^ and Piezo1^OE^ groups, characterized by intense blue staining in the tumor tissue. The quantitative data were shown in Figure 2G. This staining pattern indicated a higher degree of collagen fiber organization and deposition, which is associated with increased ECM stiffness. Taken together, the Piezo1/ITGB1 axis appeared to enhance BLCA development and progression through promoting collagen fiber accumulation and increasing ECM stiffness in vivo. These findings provided valuable insights into the molecular mechanisms underlying the role of the Piezo1/ITGB1 axis in BLCA and suggest potential therapeutic targets for intervention.

### 3.3 The Expression Levels of Piezo1/ITGB1 Axis and Its Clinical Signature

**FIGURE 3:**
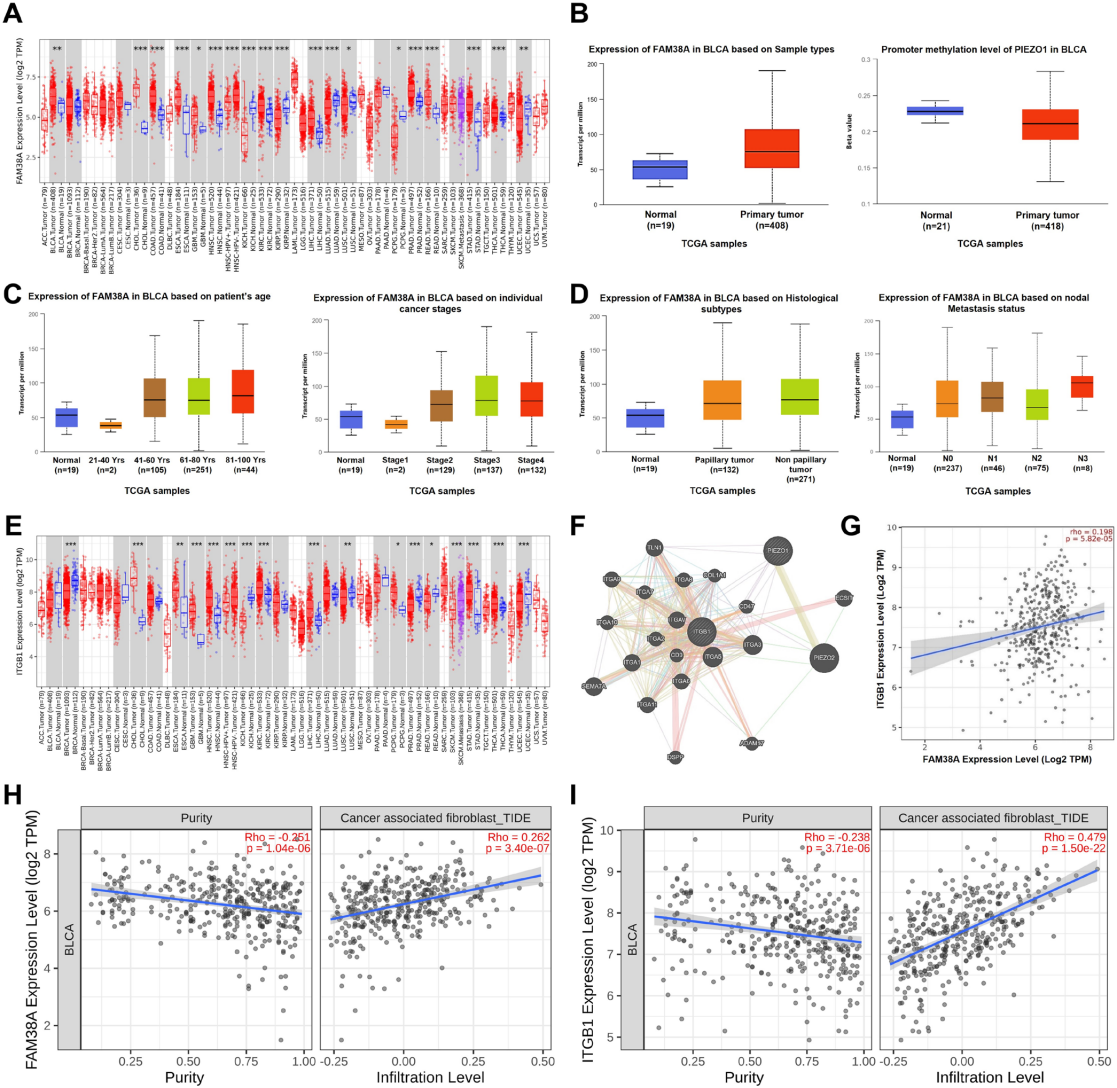
The expression level of the Piezo1/ITGB1 axis and its clinical signature. (A) Expression level of Piezo1 in pan‐cancer from TCGA data analyzed by the TIMER database. (B) Expression and methylation feature of Piezo1 from the UALCAN website. (C) Clinical signature of Piezo1 from the UALCAN website. (D) Expression of Piezo1 in BLCA based on histological subtypes and nodal metastasis status from the UALCAN website. (E) ITGB1 expression level in pan‐cancer from TCGA data analyzed by the TIMER database. (F) Interaction network between Piezo1 and ITGB1 from the GeneMINIA database. (G) The correlation between Piezo1 and ITGB1 expression levels from the TIMER website. (H) The correlation between Piezo1 and the infiltration level of CAFs in BLCA analyzed by the TIMER database. (I) The correlation between ITGB1 and the infiltration level of CAFs in BLCA analyzed by the TIMER database.

To delve deeper into the role of the Piezo1/ITGB1 axis in BLCA, we leveraged multiple databases to analyze the expression levels and clinical implications of these proteins. Our analysis of pan‐cancer data from The Cancer Genome Atlas (TCGA) using the TIMER database revealed that FAM38A, encoding Piezo1, was overexpressed in a variety of cancers, notably BLCA, cholangiocarcinoma (CHOL), kidney renal clear cell carcinoma (KIRC), and liver hepatocellular carcinoma (LIHC) (Figure 3A). The expression of FAM38A was found to be positively correlated with primary tumor, DNA methylation, patient's age, cancer stages, histological subtypes, and nodal metastasis status (Figure 3B,C, D), illustrating the obvious oncogene signature. Similarly, analysis of ITGB1 expression using the TIMER database showed overexpression in several cancers, including CHOL, esophageal carcinoma (ESCA), glioblastoma (GBM), KIRC, and LIHC (Figure 3E). However, ITGB1 expression appeared to be relatively low in BLCA, depending on the sample types and molecular subtype. The GeneMINIA database provided insights into the interaction network between Piezo1 and ITGB1, revealing that molecules such as collagen type I α1 (COL1A1), CD9, and CD47 were involved (Figure 3F). These molecules are directly connected to the ECM and the immune microenvironment, suggesting a complex interplay between the Piezo1/ITGB1 axis and the tumor microenvironment. The linear correlation between Piezo1 and ITGB1 expression levels from the TIMER website (rho = 0.198, *p* = 5.82 × 10^−5^), which was consistent with the result of tissue chips (Figure 3G). Additionally, the expression level of Piezo1 (rho = 0.262, *p* = 3.40 × 10^−7^) and ITGB1 (rho = 0.479, *p* = 1.50 × 10^−22^) was also positively related to the infiltration level of CAFs in BLCA, which was analyzed by the TIMER database (Figure 3H,I). These findings suggest that the Piezo1/ITGB1 axis may modulate each other's expression and promote BLCA progression through interactions with the ECM and CAFs. However, further external validation is needed to confirm these relationships.

### 3.4 Piezo1/ITGB1 Axis Synergistically Enhances ECM Interaction and Organization

**FIGURE 4:**
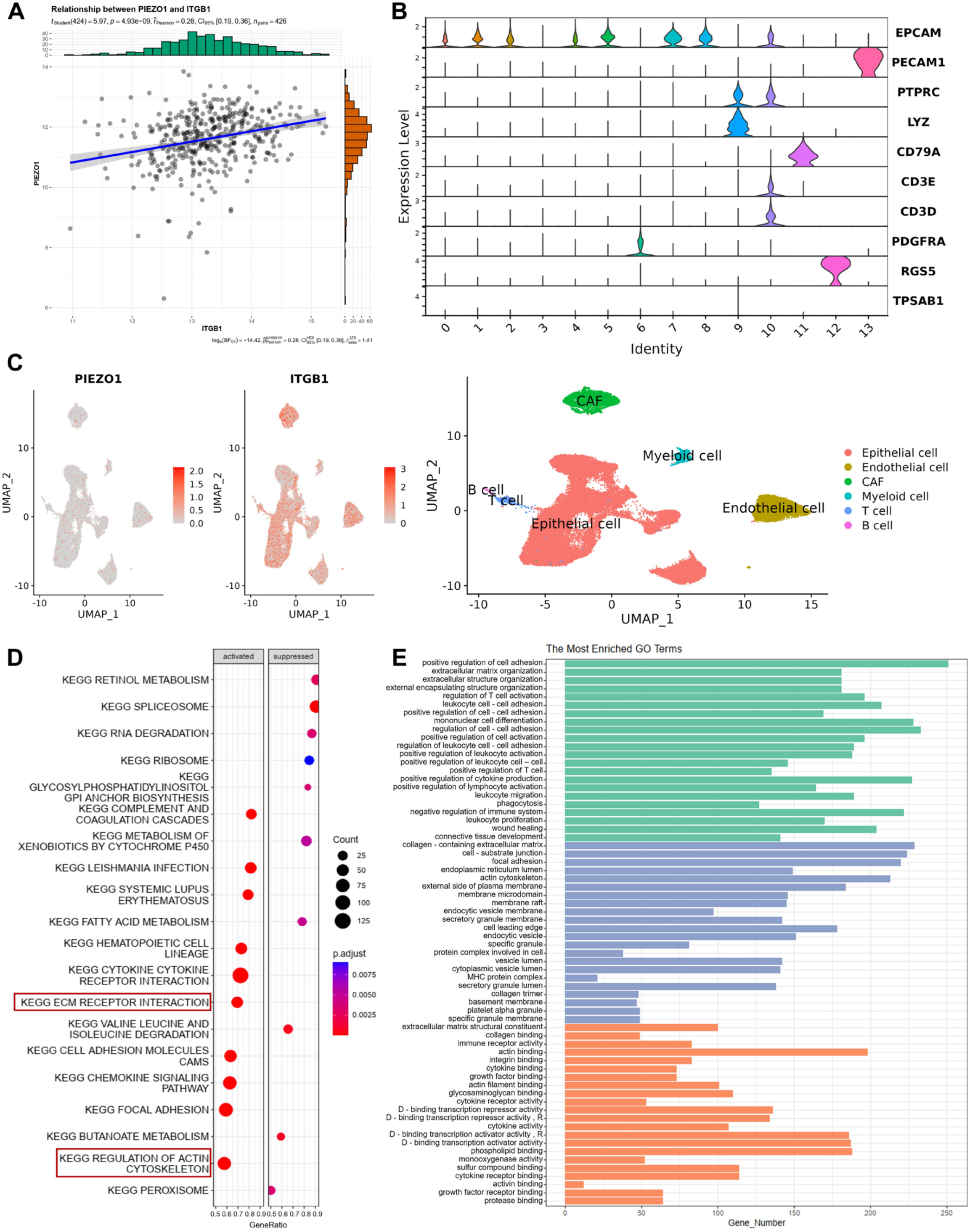
Expression profile of Piezo1/ITGB1 at the single‐cell transcriptomic level. (A) The correlation between Piezo1 and ITGB1 in BLCA according to the single‐cell sequencing. (B) Cell population annotation by the UMAP dimension reduction method according to the expression of marker genes. (C) UMAP visualization showing the expression distribution of PIEZO1 and ITGB1 at single‐cell resolution, alongside annotated cell types. (D) KEGG enrichment analysis of the Piezo1/ITGB1 axis using the R package “clusterProfiler.” (E) GO enrichment analysis of the Piezo1/ITGB1 axis using the R package “clusterProfiler.”

To gain a deeper understanding of the Piezo1/ITGB1 axis in BLCA, we analyzed single‐cell RNA sequencing (scRNA‐seq) data from the GEO database (accession number GSE135337). This dataset comprises seven BLCA samples and one pericarcinomatous sample. Utilizing the “Seurat” R package, we performed quality control on the scRNA‐seq data, excluding low‐quality cells and genes to ensure accurate analysis. There still exhibited a linear correlation between Piezo1 and ITGB1 in BLCA according to the single‐cell sequencing (Figure 4A) (*p* = 4.93 × 10^−9^, rho = 0.28, *n* = 426). To further characterize the cellular context of Piezo1 and ITGB1 expression, we employed the Uniform Manifold Approximation and Projection (UMAP) dimensionality reduction method. This approach allowed us to annotate various cell populations based on the expression of marker genes, identifying 13 distinct cell types, including cancer‐associated fibroblasts (CAFs), epithelial cells, endothelial cells, B cells, T cells, and others (Figure 4B; Figure S1). Notably, the Piezo1/ITGB1 axis was predominantly localized in CAFs and epithelial cells, suggesting a key role in establishing the relationship between the tumor and the ECM (Figure 4C). Functional enrichment analysis using the KEGG and GO databases provided insights into the biological processes modulated by the Piezo1/ITGB1 axis. KEGG analysis indicated that this axis was involved in ECM receptor interaction, focal adhesion, the PI3K‐Akt signaling pathway, and the actin cytoskeleton (Figure 4D; Figure S2). GO analysis further revealed that the Piezo1/ITGB1 axis promotes cell adhesion, ECM organization, collagen binding, and actin activity (Figure 4E). These findings collectively illustrate that the Piezo1/ITGB1 axis synergistically enhances ECM interaction and organization, thereby contributing to cancer development and progression in BLCA.

### 3.5 Piezo1/ITGB1 Promotes Proliferation and Suppresses Apoptosis in BLCA

**FIGURE 5:**
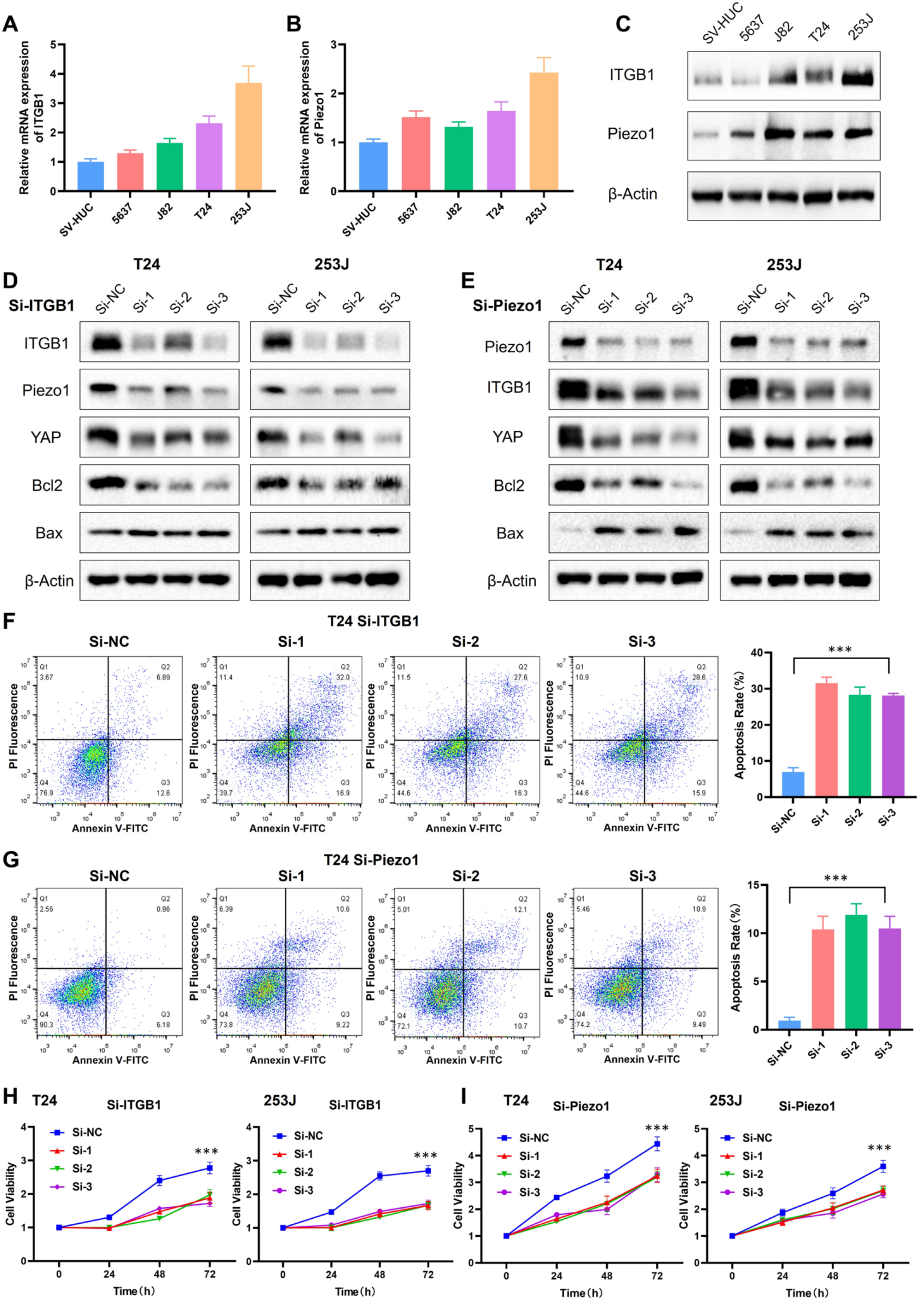
Piezo1/ITGB1 axis promotes proliferation and suppresses apoptosis in BLCA cells. (A) The expression level of ITGB1 and Piezo1 in BLCA cell lines using qRT‐PCR. (B) The expression level of Piezo1 in BLCA cell lines using qRT‐PCR. (C) Expression level of Piezo1/ITGB1 from western blotting. (D) The expression level of ITGB1/Piezo1/YAP and apoptosis‐related proteins Bcl‐2/Bax in T24 and 253J cell lines knocked down ITGB1. (E) The expression level of proteins in T24 and 253J cell lines knocked down Piezo1. (F) Apoptosis rate and quantitative data from flow cytometry in T24 cells knocked down ITGB1. (G) Apoptosis rate and quantitative data from flow cytometry in T24 cells knocked down Piezo1. (H) Cell viability and proliferation rate of T24 and 253J when ITGB1 was knocked down. (I) Cell viability and proliferation rate of T24 and 253J when Piezo1 was knocked down.

To elucidate the molecular mechanisms by which Piezo1 and ITGB1 influence BLCA, we conducted a series of in vitro experiments. As shown in Figure 5A, qRT‐PCR results indicated that the expression level of ITGB1 and Piezo1 in BLCA cell lines (5637, J82, T24, 253J) was higher than that in normal urothelial cells (SV‐HUC). Consistent with the mRNA expression data, western blot analysis confirmed that the protein levels of Piezo1 and ITGB1 were also elevated in BLCA cell lines (Figure 5C), which was also consistent with the study reported previously [27, 28].

To further investigate the functional implications of Piezo1/ITGB1 overexpression, we utilized si‐RNA to knock down the expression of Piezo1 and ITGB1 in T24 and 253J cell lines. The knockdown efficiency was validated by Western blot, showing a significant reduction (Figure 5D,E). Piezo1 was also suppressed when ITGB1 was knocked down and vice versa, meaning the expression of Piezo1 and ITGB1 could be modulated by each other and form a positive feedback loop. Given the established role of YAP in mechanotransduction and its regulation by Piezo1, we hypothesized that YAP might be a downstream effector of the Piezo1/ITGB1 axis in BLCA. Indeed, knockdown of Piezo1 or ITGB1 resulted in decreased YAP expression, further supporting the notion that YAP is involved in the mechanotransduction pathway regulated by Piezo1 and ITGB1. Besides, the expression level of apoptosis‐related proteins Bax/Bcl‐2 was also activated by si‐Piezo1/ITGB1. Then flow cytometry was applied to explore the apoptosis rate after the Piezo1/ITGB1 axis was suppressed. The results emphasized that knockdown of ITGB1 and Piezo1 increased the rate of apoptosis in T24 cells (Figure 5F,G), indicating that Piezo1 and ITGB1 could cooperate with each other to suppress apoptosis in BLCA. The impact on cell proliferation was assessed using cell viability assays. The results indicated that knockdown of Piezo1 or ITGB1 led to a significant decrease in cell viability and proliferation rate in T24 and 253J cells, suggesting that the Piezo1/ITGB1 axis promotes cell proliferation in BLCA (Figure 5H,I).

Taken together, the Piezo1/ITGB1 axis played a critical role in promoting cell proliferation and inhibiting apoptosis in BLCA, potentially through the regulation of the YAP signal. These results highlight the therapeutic potential of targeting the Piezo1/ITGB1 axis in the treatment of cancer.

### 3.6 Piezo1/ITGB1 Activates Ca^2+^/YAP Signal in BLCA Under Different Stimulation Conditions

**FIGURE 6:**
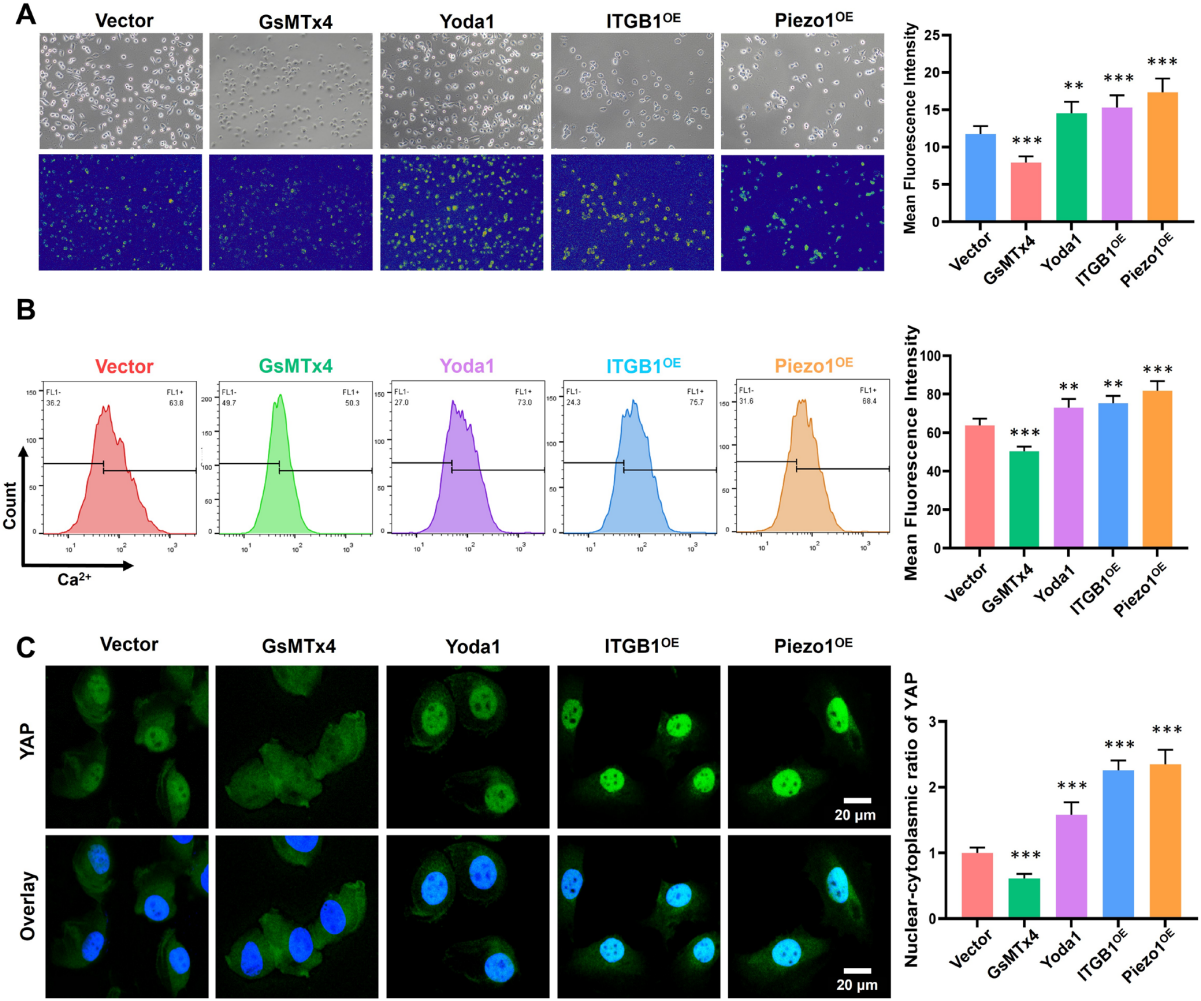
Piezo1/ITGB1 activates Ca^2+^ influx and YAP signal in BLCA cells under different stimulations. (A) The fluorescence image and quantitative analysis of Ca^2+^ signal using Fluo‐4 AM probe in Piezo1^OE^, ITGB1^OE^, and Vector groups treated with Yoda1 or GsMTx4. (B) The flow cytometry results and quantitative analysis of Ca^2+^ signal in various cells. (C) The immunofluorescence image and quantitative analysis of YAP signaling in different cells.

The Piezo1/ITGB1 axis, known for its role in mechanotransduction, also plays a crucial role in mediating the release of calcium ions (Ca^2+^) and initiating downstream signaling pathways [29]. The influx of Ca^2+^ is indicative of Piezo1 activation, and thus, we aimed to investigate how this axis modulates Ca^2+^ signaling in cells under various conditions. We utilized a Fluo‐4AM fluorescence probe, specific agonists (Yoda1) and inhibitors (GsMTx4) of Piezo1 to analyze the Ca^2+^ signal in cells. As shown in Figure 6A, the Piezo1^OE^ and ITGB1^OE^ groups exhibited a significantly stronger and more robust Ca^2+^ signal compared to the Vector control group. The Yoda1 treatment led to a noticeable strengthening of the Ca^2+^ signal, confirming that Piezo1 activation enhances Ca^2+^ release. Conversely, treatment with GsMTx4 weakened the Ca^2+^ signal in both Piezo1^OE^ and ITGB1^OE^ cells, indicating that Piezo1 inhibition reduced Ca^2+^ influx. To corroborate the fluorescence findings, we employed flow cytometry to measure the Ca^2+^ signal in cells under various conditions (Figure 6B). The results were consistent with the fluorescence assay, showing a positive correlation between Piezo1/ITGB1 expression and Ca^2+^ release. The modulation of Piezo1 activity through agonists and inhibitors respectively enhanced and diminished the Ca^2+^ signal, further supporting the role of Piezo1/ITGB1 in Ca^2+^ homeostasis in BLCA cells.

Immunofluorescence assay was utilized to further investigate the correlation between Ca^2+^ and YAP signaling. The Piezo1/ITGB1 axis promoted the release of Ca^2+^ influx, which in turn enhanced YAP signaling and its translocation into the nucleus (Figure 6C). In conclusion, our study demonstrated that the Piezo1/ITGB1 axis was a critical mediator of Ca^2+^ influx in BLCA. The activation of this axis, either through overexpression or stimulation with agonists, led to an increase in Ca^2+^ and YAP signaling and its translocation into the nucleus, which was a key event in the cellular response to mechanical stimuli. Activation of Ca^2+^/YAP can lead to the transcription of related genes that promote cell proliferation and survival.

### 3.7 ECM Stiffness Promotes Proliferation Through Activating Piezo1/ITGB1 Axis and YAP Signal

**FIGURE 7:**
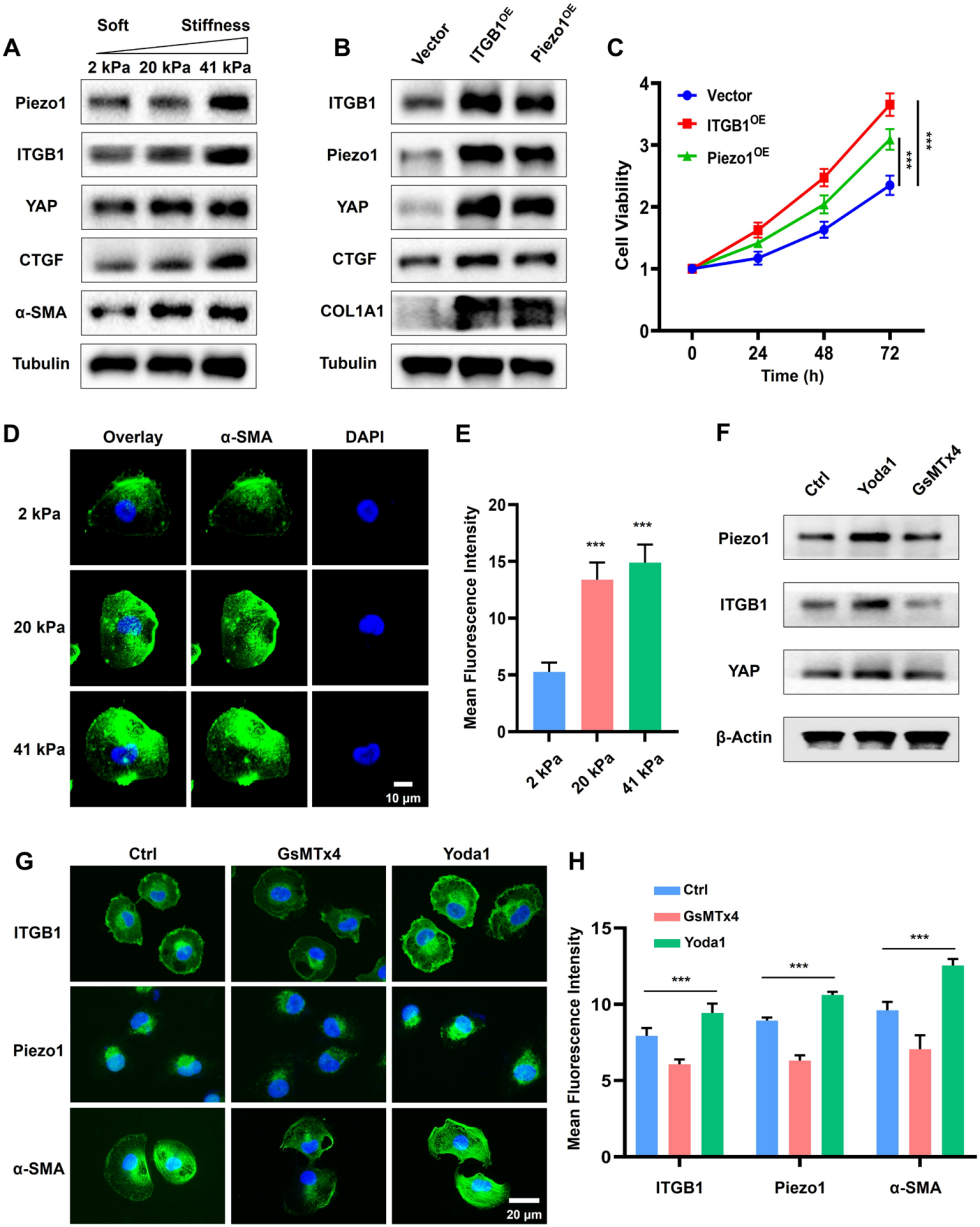
Activation of the Piezo1/ITGB1 axis promotes BLCA proliferation and ECM stiffness. (A) Protein expression in T24 cells on different stiffness substrates (2, 20, 41 kPa hydrogel). (B) The protein expression level in Piezo1^OE^ and ITGB1^OE^ cell lines. (C) Cell viability and proliferation rates in Piezo1^OE^ and ITGB1^OE^ cell lines. (D) Expression level of α‐SMA in different stiffness substrates from the immunofluorescence assay. (E) The quantitative analysis of fluorescence intensity of α‐SMA. (F) Expression level of Piezo1/ITGB1/YAP proteins from western blotting when the agonists (Yoda1) and inhibitors (GsMTx4) of Piezo1 were used. (G) Fluorescence image of Piezo1/ITGB1/α‐SMA proteins when Yoda1 and GsMTx4 were used. (H) The quantitative analysis of fluorescence intensity of Piezo1/ITGB1/α‐SMA.

To dissect the interplay between the Piezo1/ITGB1 axis and the mechanical properties of ECM, we designed experiments to evaluate how varying degrees of ECM stiffness affect the expression of Piezo1, ITGB1, and related proteins in cells. We synthesized hydrogels with different stiffness levels (2, 20, 41 kPa) to mimic the physiological and pathological environments of the ECM [26]. Our results demonstrated that the expression levels of Piezo1, ITGB1, YAP, Connective Tissue Growth Factor (CTGF), and α‐SMA increased with the increase in matrix stiffness (Figure 7A). It has been reported that α‐SMA and CTGF promote cell proliferation, migration, and cancer development through remodeling ECM [30, 31]. This trend suggested that the Piezo1/ITGB1 axis was not only responsive to but also contributed to the establishment of ECM stiffness and its associated structural components. Overexpression of Piezo1 led to an increase in ITGB1 levels, and vice versa, reinforcing the existence of a positive feedback loop between these two proteins (Figure 7B). Additionally, the overexpression of Piezo1 and ITGB1 (Piezo1^OE^ and ITGB1^OE^) significantly upregulated YAP, CTGF, and COL1A1, key players in ECM stiffness and collagen deposition, indicating a direct role of the Piezo1/ITGB1 axis in promoting a stiff ECM environment. Besides, Piezo1^OE^ and ITGB1^OE^ also promoted cell proliferation obviously compared with the Vector group (Figure 7C).

Next, the immunofluorescence assay was utilized to observe the expression of α‐SMA, a marker of myofibroblast differentiation and ECM remodeling, under different stiffness conditions. The expression of α‐SMA and actin microfilaments increased with ECM stiffness (Figure 7D), indicating a structural adaptation of cells to the mechanical environment. Quantitative analysis of fluorescence changes was presented in Figure 7E. To further validate the connection between Piezo1/ITGB1 and α‐SMA, the agonists (Yoda1) and inhibitors (GsMTx4) of Piezo1 were utilized. The expression level of Piezo1/ITGB1/YAP proteins was activated and suppressed by Yoda1 and GsMTx4, separately (Figure 7F). Additionally, GsMTx4 suppressed the Piezo1/ITGB1/α‐SMA signaling, while Yoda1 enhanced it, demonstrating that Piezo1 activity is a critical regulator of α‐SMA expression and actin cytoskeleton organization (Figure 7G). The quantitative analysis of fluorescence changes was presented in Figure 7H. These results underscored the role of the Piezo1/ITGB1 axis in promoting cell growth, potentially through modulating and involving the ECM. In summary, our findings indicated that ECM stiffness activated the Piezo1/ITGB1/YAP axis, which in turn further strengthened ECM accumulation and promoted cell proliferation, forming a positive feedback loop that drove the progression of cancer.

## 4 Discussion

**FIGURE 8:**
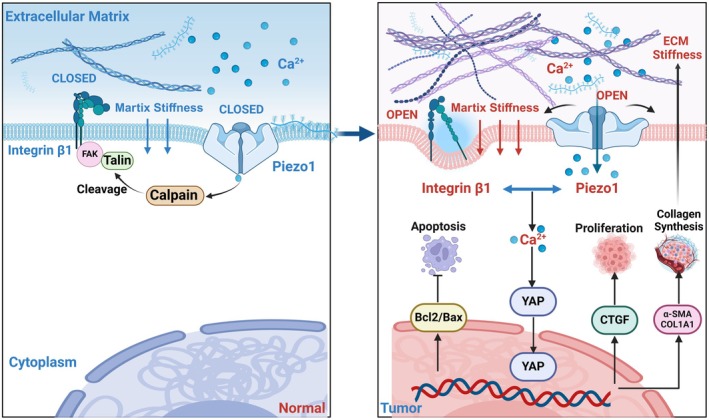
Piezo1/ITGB1 activation and Ca^2+^/YAP pathway participate in ECM stiffness‐induced proliferation and development in BLCA.

This study presents a comprehensive analysis of the Piezo1/ITGB1 axis in BLCA, revealing its significant role in modulating ECM stiffness and collagen accumulation. Our findings underscore the importance of this axis in both cellular and tissue‐level processes, highlighting its impact on tumor progression and patient prognosis. The Piezo1/ITGB1 axis significantly enhances ECM stiffness and the accumulation of collagen, which is a critical factor in the tumor microenvironment that can promote cancer cell behavior, including proliferation, migration, and invasion. The Piezo1/ITGB1 axis forms a positive feedback loop with matrix stiffness, amplifying the mechanosensory capacity of tumor cells and promoting their proliferation and progression (Figure 8). By disrupting this loop, it may be possible to attenuate the aggressive behavior of BLCA and improve patient outcome.

The healthy bladder wall is a mechanically inhomogeneous tissue, with a gradient of increasing stiffness from the urothelium to the lamina propria, which gradually decreases when reaching the muscle outer layer. An increase in tissue compliance is observed before the onset and invasion of the tumor [11]. It has also been found that stiffness changes in the different mucosa layers can result in aberrant tissue morphologies. The resulting thickening, wrinkles, and folds resemble early papillary tumors and carcinomas in situ [32]. Collagen stiffness has been proposed to promote BC progression from NMIBC to MIBC and induced cell proliferation, EMT, and favored the migration of the invasion front of the tumor [13]. The mechanosensitive ion channel Piezo1 and ITGB1 have emerged as critical players in the mechanotransduction pathways that link ECM stiffness to intracellular signaling. Piezo1 is uniquely positioned to sense mechanical forces associated with ECM stiffening and translate these forces into biochemical signals, notably through the modulation of Ca^2+^ signaling [33]. The extracellular domain of ITGB1 connects the ECM to the cytoskeleton, allowing cells to sense and respond to mechanical forces and Ca^2+^‐signaling through assembling cytoplasmic complexes [34, 35, 36, 37]. There is a growing recognition of the interplay between Piezo1 activation and integrin‐related signaling. For instance, in the context of kidney fibrosis, increased tissue stiffness activates Piezo1, leading to Ca^2+^ influx, which promotes ITGB1 clustering and ECM accumulation [19]. Moreover, excessive deposition of ECM in turn increases Piezo1 expression and enhances the mechanosensory capacity, aggravating the progression of renal fibrosis. Xu et al. also reported a mechanical positive feedback loop between ITGB1 and Piezo1 activation that forms a bistable switch to modulate downstream signaling involving Ca^2+^ and YAP, recursively leading to cardiac fibroblast phenotype [26, 38]. Massimo et al. demonstrated that α5β1 integrin was identified as a specific marker in 81% of human high‐grade NMIBC and used as a target for the delivery of gold nanorods and aptamers [39, 40]. Our study is the first to delineate the role and mechanism of Piezo1/ITGB1 in BLCA progression. Targeting this axis could potentially disrupt the positive feedback loop that drives ECM stiffening and cancer progression, which offers a new avenue for the development of more effective treatments.

The role of intracellular Ca^2+^ in cancer biology is multifaceted, influencing a range of cellular processes that are critical to tumor progression, including increased proliferation, angiogenesis, migration, invasion, and so on [41, 42, 43]. Ca^2+^ local influx at focal adhesion promotes integrin complex assembly, FAK signaling activation, ECM remodeling, and further upregulates Piezo1 in cells [44, 45, 46, 47]. Our study contributes to the understanding of how Ca^2+^ signaling is intertwined with the Piezo1/ITGB1 axis in BLCA, and reveals a complex interplay between the Piezo1/ITGB1 axis, Ca^2+^ signaling, and ECM remodeling in BLCA. The Piezo1/ITGB1 axis activates the release of Ca^2+^ influx, which in turn enhances YAP signaling and its translocation into the nucleus, promoting ECM remodeling and tumor stiffness. This feedback loop between mechanotransduction and tissue stiffness is a critical driver of cancer development.

The tumor microenvironment is a complex and dynamic landscape that plays a crucial role in cancer progression, resulting in excessive deposition and crosslinking of ECM components such as collagen, fibronectin, etc. [48, 49]. Then, increased ECM deposition promotes ECM stiffness, which also activates α‐SMA phenotypes [50]. High ECM stiffness is associated with increased tumor cell proliferation and survival. It also enhances tumor cell stemness, antiapoptotic and self‐renewal capabilities, migration, and drug resistance. These properties contribute to the malignant potential of cancer and their ability to invade and metastasize [51]. Our study demonstrates that the Piezo1/ITGB1 axis enhances the rapid proliferation and antiapoptotic capabilities of tumor cells through enhancing Ca^2+^ and modulating key proteins such as YAP, α‐SMA, and so on. These proteins are central to the mechanotransduction pathways that allow cells to sense and respond to mechanical cues from the ECM, eventually promoting the development of cancer.

The specific Piezo1 inhibitor GsMTx4 has demonstrated preliminary efficacy in preclinical studies, where it suppresses the self‐renewal capacity of cancer stem cells and induces apoptosis by blocking Piezo1‐mediated calcium signaling, ultimately prolonging survival in murine models [52]. Additionally, blockade of Piezo1 in T cells has been shown to significantly enhance their cytotoxic activity against tumor cells [53]. Meanwhile, several integrin inhibitors targeting integrin‐associated signaling pathways have entered clinical trials, exhibiting potential to overcome therapeutic resistance and improve treatment outcomes [7]. The synergistic potential between Piezo1 inhibition and integrin‐targeted agents could address tumor microenvironment‐driven resistance mechanisms, particularly in malignancies where ECM stiffness and mechanosensitive pathways drive progression [54]. However, challenges such as optimizing inhibitor specificity, minimizing off‐target effects in normal tissues, and developing efficient delivery systems for clinical translation require further investigation.

## 5 Conclusion

Our study uncovers a synergistic mechanotransduction pathway in which the Piezo1/ITGB1 axis and Ca^2+^/YAP signaling cooperatively drive BLCA progression via ECM stiffness sensing. Reciprocal regulation between Piezo1 and ITGB1 establishes a clinically adverse profile, correlating with poor OS, PFS, RFS, and elevated ECM rigidity in vivo. Mechanistically, Piezo1/ITGB1 activation triggers Ca^2+^ influx, which facilitates nuclear translocation of YAP signal, upregulating α‐SMA, COL1A1, and enhancing collagen deposition. This ECM remodeling further amplifies matrix stiffness, creating a self‐reinforcing loop that sustains Piezo1/ITGB1 hyperactivity, perpetuates YAP‐driven proliferation, and suppresses apoptosis. The interdependence of Piezo1/ITGB1 activation and ECM stiffening highlights a feedforward mechanism that accelerates BLCA aggressiveness, proposing dual targeting of mechanosensitive pathways and ECM dynamics as a promising therapeutic strategy.

## Author Contributions

**Minghai Ma:** conceptualization; data curation; formal analysis; investigation; methodology; writing – original draft; writing – review and editing. **Jianpeng Li:** data curation; formal analysis; methodology; validation; writing – review and editing. **Xing Li:** data curation; investigation; methodology. **Minxuan Jing:** formal analysis; supervision; validation. **Lu Wang:** data curation; investigation; methodology. **Yunzhong Jiang:** investigation; methodology; resources. **Zezhong Yang:** investigation; methodology. **Jiale He:** formal analysis; resources. **Min Wang:** formal analysis; resources. **Hang Liu:** formal analysis; resources. **Yutong Chen:** formal analysis; resources. **Kaibo Mi:** formal analysis; resources. **Lei Wang:** conceptualization; funding acquisition; investigation; project administration; supervision; writing – review and editing. **Jinhai Fan:** conceptualization; funding acquisition; investigation; project administration; supervision. **Hongxia Du:** conceptualization; investigation; project administration; supervision; writing – review and editing.

## Ethics Statement

The study was conducted in accordance with the Declaration of Helsinki and approved by the Ethical Committee of First Affiliated Hospital of Xi'an Jiaotong University (Approval Number: LLSBPJ‐2023‐095). All the methods were carried out according to the relevant guidelines and regulations. All the animal experimental protocols were approved by the Experimental Animal Ethics Committee of Xi'an Jiaotong University (Approval Number: XJTUAE2023‐545).

## Conflicts of Interest

The authors declare no conflicts of interest.

## Supporting information


Data S1.

## Data Availability

Data will be made available on request.
