# Supplementary material for: Piezo1/ITGB1 Synergizes With Ca2+/YAP Signaling to Propel Bladder Carcinoma Progression via a Stiffness‐Dependent Positive Feedback Loop
Source: Cancer Med. 2025 Jul 16;14(14):e71059. doi: 10.1002/cam4.71059 (PMC12264578; doi:10.1002/cam4.71059)
Supplement: Supplementary file 1 — Data S1. [file CAM4-14-e71059-s001.docx]

**Piezo1/ITGB1 Synergizes with Ca²⁺/YAP Signaling to Propel Bladder Carcinoma Progression via a Stiffness-Dependent Positive Feedback Loop**

Minghai Ma^2, 3+^, Jianpeng Li^2+^, Xing Li^3^, Minxuan Jing^2^, Lu Wang^2^, Yunzhong Jiang^2^, Zezhong Yang^2^, Jiale He^2^, Min Wang^2^, Hang Liu^2^, Yutong Chen^2^, Kaibo Mi^2^, Lei Wang^3^, Jinhai Fan^2*^, and Hongxia Du^1*^

1 Department of Pharmacology and Toxicology, School of Basic Medical Sciences, Xi'an Medical University, Xi'an 710021, China.

2 Department of Urology, The First Affiliated Hospital, Xi’an Jiaotong University, Xi’an 710061, China.

3 Department of Thoracic Surgery, Tangdu Hospital, Air Force Medical University, Xi’an 710038, China.

^*^Correspondence:

fanjinhai@xjtu.edu.cn; duhongxia@xiyi.edu.cn.

^+^ These authors contributed equally to this work.

**Table S1.** The Gene-specific primers used in the study.

| ITGB1 Reverse | CCTTTGCTACGGTTGGTTACATT |
| --- | --- |
| ITGB1 Forward | CCTACTTCTGCACGATGTGATG |
| Piezo1 Reverse | GGGCACAATATGCAGGCAGA |
| Piezo1 Forward | GGACTCTCGCTGGTCTACCT |
| 18S Reverse | TAGTAGCGACGGGCGGTGTG |
| 18S Forward | CAGCCACCCGAGATTGAGCA |

**Table S2.** The chemicals and antibodies used in the study.

| REAGENT | SOURCE | IDENTIFIER |
| --- | --- | --- |
| Antibodies | | |
| Piezo1 antibody | Abclonol | A23380 |
| Integrin β1 | Abclonol | A22599 |
| YAP1 | Abclonol | A1002 |
| α-SMA | Abcam | ab7817 |
| COL1A1 | Abclonol | A1352 |
| Bcl2 | Abcam | ab32124 |
| Bax | Abcam | ab182734 |
| β-Actin | Abclonol | AC038 |
| CTGF | Abclonol | A11067 |
| α-Tubulin | Abclonol | AC012 |
| Chemicals | | |
| Yoda1 | MedChemExpress (MCE) | HY-18723 |
| GsMTx4 | MedChemExpress (MCE) | HY-P1410 |
| Integrin β1 plasmids | Biokeeper | N/A |
| Piezo1 plasmids | Biokeeper | N/A |
| Piezo1 si-RNA | Genepharma Technology | N/A |
| Integrin β1 si-RNA | Genepharma Technology | N/A |
| Critical commercial assays | | |
| Cell counting kit-8 (CCK8) | TargetMol | C0005 |
| FITC Annexin V Apoptosis Detection Kit | BD Pharmingen | 556547 |
| Fluo-4 AM Probe | Beyotime Biotechnology | S1060 |

**Table S3.** Association between Piezo1 and ITGB1 expression with clinicopathologic characteristics in BLCA tissues

| Variable | Overall, N = 181 | Piezo1 and ITGB1 expression | | P  value |
| --- | --- | --- | --- | --- |
|  |  | **High N = 91 (50%)** | **Low N = 90 (50%)** |  |
| Age | 69.00 [61.00, 74.00] | 69.00 [61.00, 76.00] | 68.00 [61.25, 73.00] | 0.375 |
| Gender |  |  |  | 0.830 |
| Male | 136 (75.14%) | 69 (75.82%) | 67 (74.44%) |  |
| Female | 45 (24.86%) | 22 (24.18%) | 23 (25.56%) |  |
| Tumor Size |  |  |  | 0.028 |
| ＜ 3 cm | 137 (75.69%) | 63 (69.23%) | 74 (82.22%) |  |
| ≥ 3 cm | 44 (24.31%) | 28 (30.77%) | 16 (17.78%) |  |
| Tumor Number |  |  |  | 0.037 |
| ≤ 1 | 94 (51.93%) | 38 (41.76%) | 56 (62.22%) |  |
| ≥ 2 | 87 (48.07%) | 53 (58.24%) | 34 (37.78%) |  |
| Smoking |  |  |  | 0.506 |
| 0 | 101 (55.80%) | 53 (58.24%) | 48 (53.33%) |  |
| 1 | 80 (44.20%) | 38 (41.76%) | 42 (46.67%) |  |
| Hypertension |  |  |  | 0.285 |
| 0 | 124 (68.51%) | 59 (64.84%) | 65 (72.22%) |  |
| 1 | 57 (31.49%) | 32 (35.16%) | 25 (27.78%) |  |
| Diabetes |  |  |  | 0.588 |
| 0 | 148 (81.77%) | 73 (80.22%) | 75 (83.33%) |  |
| 1 | 33 (18.23%) | 18 (19.78%) | 15 (16.67%) |  |


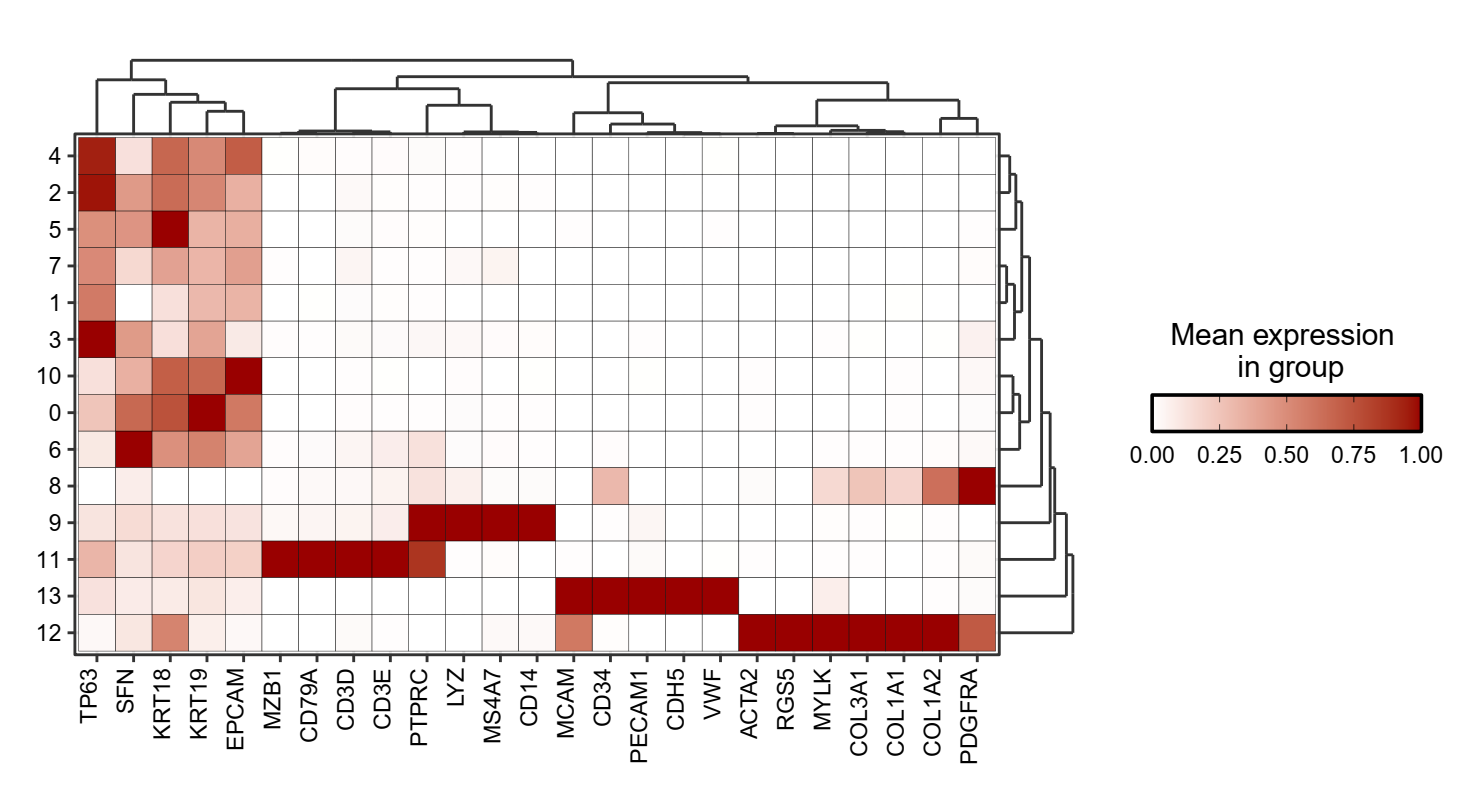


**Fig. S1** Cell population annotation by the UMAP dimension reduction method according to the expression of marker genes.

**
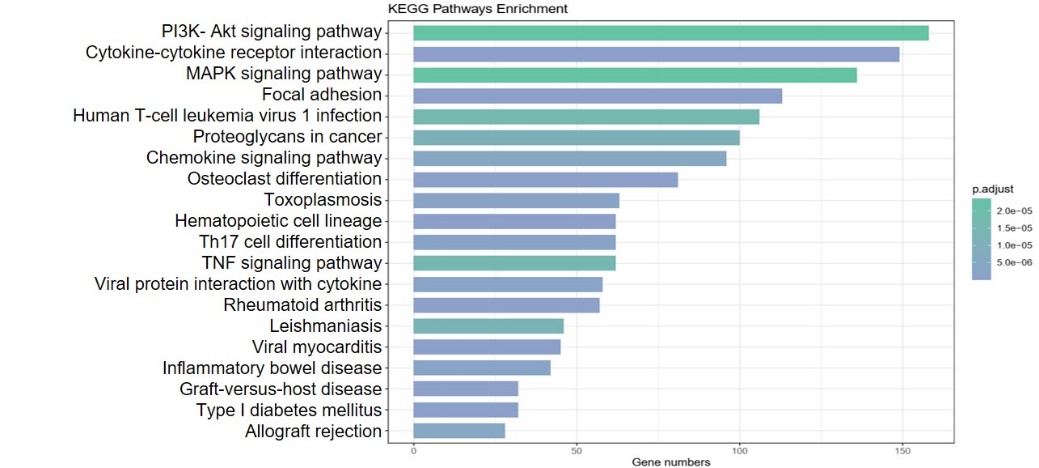
**

**Fig. S2** KEGG analysis of Piezo1/ITGB1 axis from scRNA -seq data.


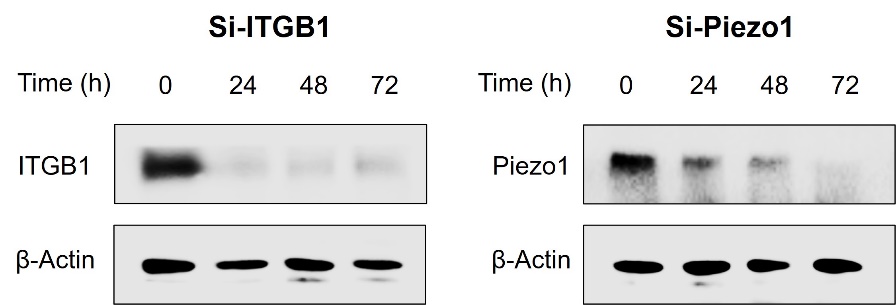


**Figure S3.** The expression level of ITGB1/Piezo1 in T24 cell knocked down ITGB1 or Piezo1 after 24, 48, and 72 h.


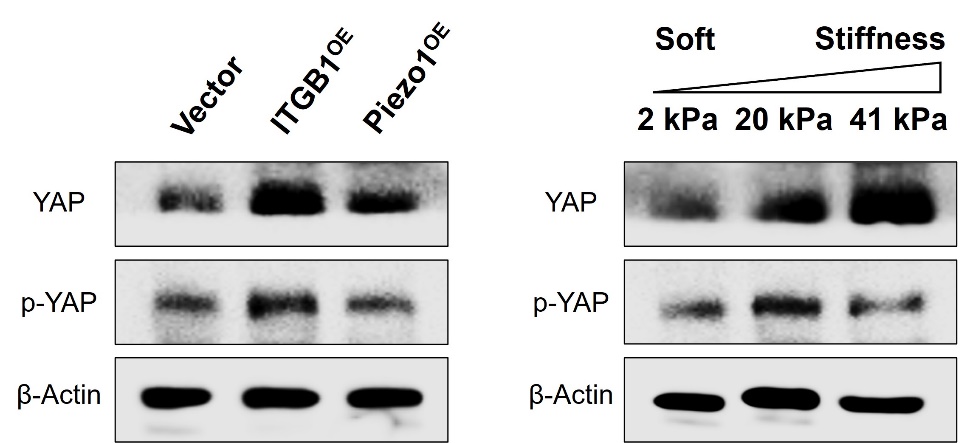


**Figure S4.** The expression level of YAP/p-YAP in T24 cell overexpressed ITGB1 or Piezo1 and treated with different stiffness.
